# Editorial: Genomic insights into sheep and goat breeding efficiency, volume II

**DOI:** 10.3389/fvets.2026.1850491

**Published:** 2026-04-29

**Authors:** Mingli Peng, Ziyang Xu, Xiao Zhang, Jiawei Wang, Rui Ding, Fei Hao

**Affiliations:** 1 Key Laboratory of Reproductive Regulation and Breeding of Grassland Livestock, School of Life Sciences, Inner Mongolia University, Hohhot, China; 2 Northumbria University, Newcastle upon Tyne, United Kingdom

**Keywords:** genetic improvement, molecular regulatory networks, multi-omics integration, quantitative traits, sheep and goat breeding

As a globally influential academic platform in the field of animal husbandry and veterinary medicine, *Frontiers in Veterinary Science* consistently focuses on cutting-edge research pertaining to genetic improvement and molecular regulation in livestock and poultry, thereby continuously fostering scholarly exchange and the translational application of research outcomes in this field. Following the successful publication of *Genomic Insights into Sheep and Goat Breeding Efficiency - Volume I*, the journal is pleased to launch Volume II, which aims to systematically synthesize the latest research advances and provide an academic platform for in-depth exchange among researchers worldwide.

Sheep and goats serve as vital livestock resources for the development of global animal husbandry (1, 2). Their economic traits, including meat, milk, and textile fibers, are typical quantitative genetic traits influenced by the combined regulation of multiple genes and environmental factors (1, 3). Although considerable progress has been made in understanding the genetic foundations of sheep and goats, critical knowledge gaps remain in areas such as precise genotype-phenotype association analysis, exploration of genomic footprints of domestication and breeding history, and the construction of molecular regulatory networks for complex economic traits (1, 4). The Research Topic aims to systematically analyze the genetic diversity and key genetic characteristics of sheep and goats, with the goal of uncovering their intrinsic genetic patterns, identifying functional genes and crucial metabolic and signaling pathways that regulate important economic traits, and establishing efficient molecular marker-assisted breeding strategies. Ultimately, livestock metabolic processes are optimized and production efficiency is enhanced, thereby accelerating the breeding of high-quality sheep and goat varieties with superior reproductive performance, strong stress resistance, and outstanding production traits (5–7).

The 11 representative research papers featured in this Research Topiccollectively and precisely respond to the aforementioned objectives, comprehensively presenting the latest advances in the field across multiple dimensions—namely, the elucidation of gene functions, the regulatory roles of non-coding RNAs, genetic variation association mapping, and multi-omics integration. Regarding morphology and environmental adaptability, Mei et al. conducted an in-depth investigation focusing on keratin 1 (KRT1), demonstrating that KRT1 regulates horn development in sheep through the coordinated action of tissue-specific expression, protein–protein interaction stability, and genetic variation, thereby offering potential molecular markers for horn-type breeding. Concurrently, Yang et al. systematically analyzed the adaptive mechanisms of Ganxi goats to humid and hot environments, revealing the adaptive characteristics of their skin structure and the potential role of the GSDMA protein in regulating the hair follicle cycle, further enriching our understanding of the genetic mechanisms underlying the adaptability of local goat breeds.

In the field of production performance enhancement, substantial research achievements have been made, with breakthrough progress realized in meat production and wool/cashmere production. In terms of meat traits, Han et al. systematically reviewed the relevant research findings from 2018 to 2025, integrating technical approaches including genome-wide association studies, transcriptome sequencing, and candidate gene analysis, identifying key candidate genes such as *VRTN, MSTN, and DGAT1* that regulate the number of sheep vertebrae, body growth, muscle development, and fat deposition, and successfully established a genetic marker resource library for sheep meat traits. Meanwhile, Chen et al. conducted innovative research on the gut-muscle regulatory axis. Using transcriptome sequencing and metabolite profiling, they mapped the segment-specific transcriptome profile of the sheep gastrointestinal tract for the first time, elucidating how gene expression in distinct intestinal segments regulates muscle amino acid composition, fatty acid metabolism, and the synthesis of flavor precursors. In terms of wool and cashmere production traits, Wu et al. employed transcriptome sequencing to identify the key microRNA (oar-miR-370-3p) regulating the fiber diameter of Ordos fine-wool sheep, elucidating its mechanism of action involving targeted regulation of fibroblast proliferation. Chunhua et al. integrated multi-omics technologies with artificial intelligence-based analytical approaches to systematically dissect the regulatory network of miRNAs in cashmere goat hair follicle development, thereby providing precise molecular targets and technical pathways for cashmere quality improvement. Gao et al. conducted studies on secondary hair follicle-derived dermal papilla cells (SHF-DPCs) in cashmere goats, demonstrating that all-trans retinoic acid (ATRA) regulates their proliferation and apoptosis via the TGF-β2/Smad2/3 signaling pathway, offering novel mechanistic insights for molecular improvement of cashmere yield and quality.

Given that enhancing reproductive efficiency is a central objective in sheep and goat breeding, the studies presented in this Research Topic provide novel academic perspectives for further exploration in this field. Xuan et al. performed transcriptome sequencing of hypothalamic tissues at different developmental stages in Duolang sheep, revealing that lncRNAs are involved in the regulation of estrus initiation through ceRNA networks and pathways such as GnRH, leading to the identification of the core regulatory factors involved in this process. Muhetaer et al. analyzed the differential expression patterns of lncRNAs and mRNAs in ovarian tissues during estrus in Pishan Red Sheep and Hu Sheep with different FecB genotypes, constructed related regulatory networks, and identified key molecular candidates including *GDF9* and *MSTRG.61044.1*. Liu et al. performed CNV-based genome-wide association studies in Inner Mongolia cashmere goats, uncovering that genes such as *ZN845, SOX15, and FGF11* regulate early growth traits, providing valuable genetic resources for the molecular breeding of growth traits in goats. Furthermore, Lian et al. demonstrated that quercetin regulates the proliferation and apoptosis of secondary hair follicle stem cells in cashmere goats by activating signaling pathways such as PI3K–Akt, Wnt, and TGF-β, offering robust experimental support for the development of novel technical strategies to enhance cashmere yield.

The research findings presented in this Research Topic possess both theoretical and practical application value, encompassing original discoveries such as the dissection of core genes and the construction of regulatory networks, as well as technological innovations including multi-omics integration and AI-assisted analysis. Together, they form a complete research chain from the elucidation of molecular mechanisms to the application of breeding technologies, effectively filling critical knowledge gaps in the field of sheep and goat breeding. These achievements not only advance theoretical innovation in sheep and goat genomics but also provide essential guidance for precision breeding practices in the global livestock industry, further deepening the scientific community's understanding of the genetic basis underlying key economic traits in livestock and providing a solid foundation for the implementation of related breeding technologies.

*Frontiers in Veterinary Science* will continue to serve as a global platform for high-level academic exchange, and we anticipate that the contents of this volume will provide valuable references for researchers in the field, supporting studies on genetic improvement of sheep and goats and the high-quality development of the industry, thereby promoting the coordinated development of global livestock through breed optimization, quality enhancement, and ecological sustainability.
